# Supplementary figures and images for: Vav1 and mutant K-Ras synergize in the early development of pancreatic ductal adenocarcinoma in mice
Source: Life Sci Alliance. 2020 Apr 10;3(5):e202000661. doi: 10.26508/lsa.202000661 (PMC7156281; doi:10.26508/lsa.202000661)

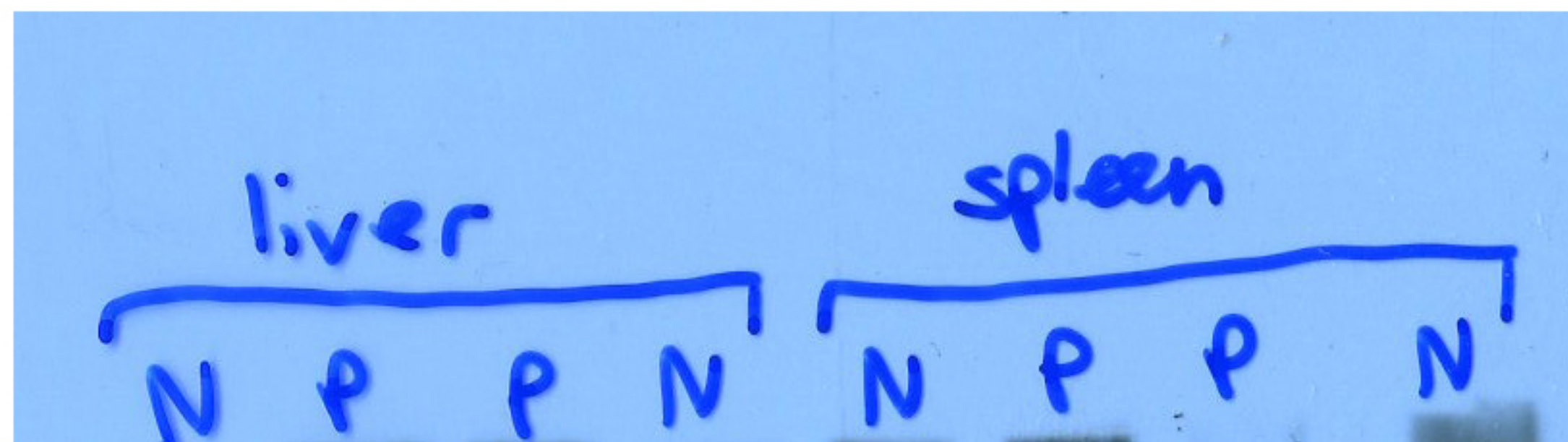

26FP

120  
95  
78

1  
1  
1  
1

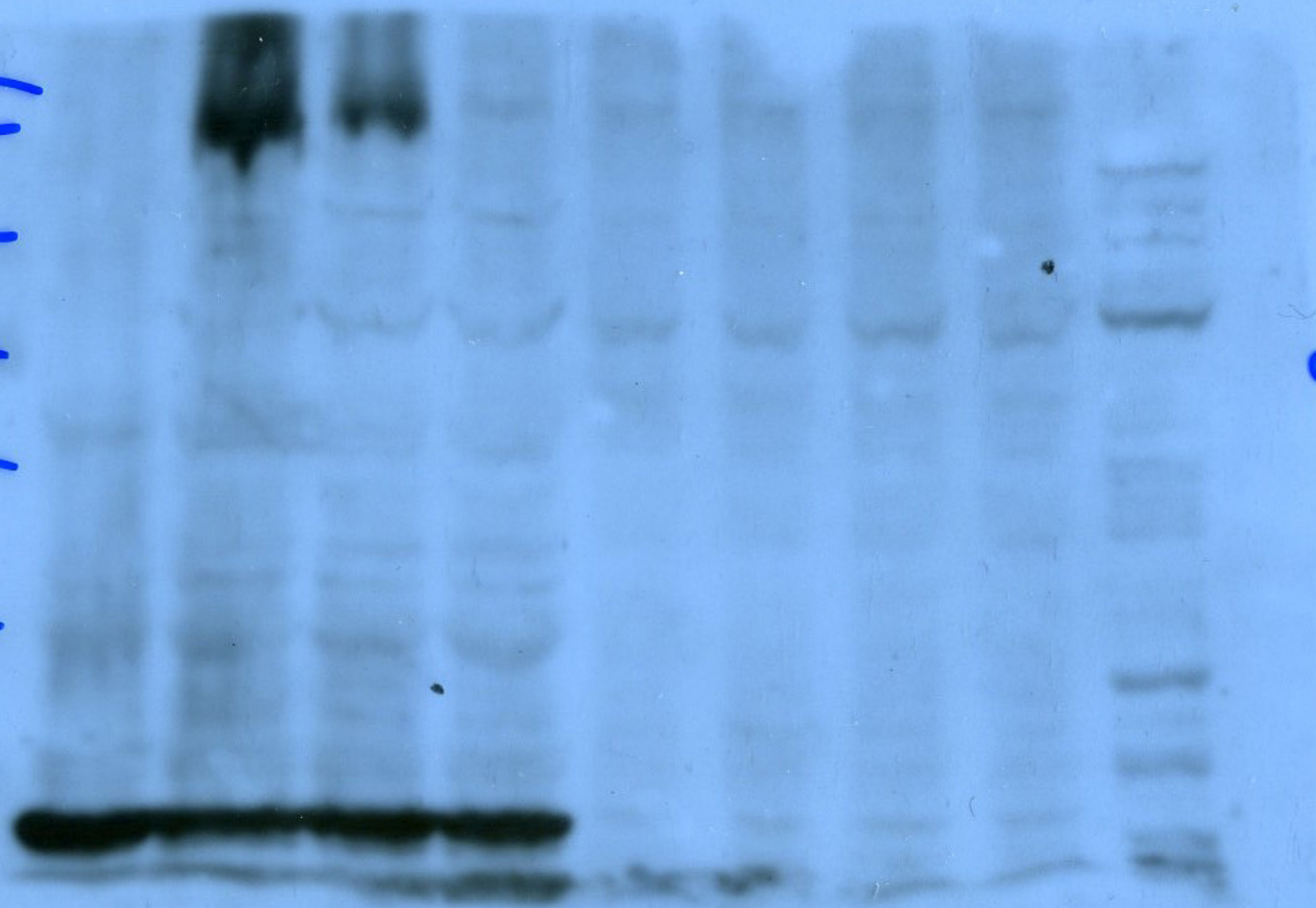

19.01.12

Supplement: Supplementary file 1 [file LSA-2020-00661_SdataFS2_1.pdf]

Liver spleen

N P P N N P P N

GFP ←

78 —  
—  
—  
—  
—  
—

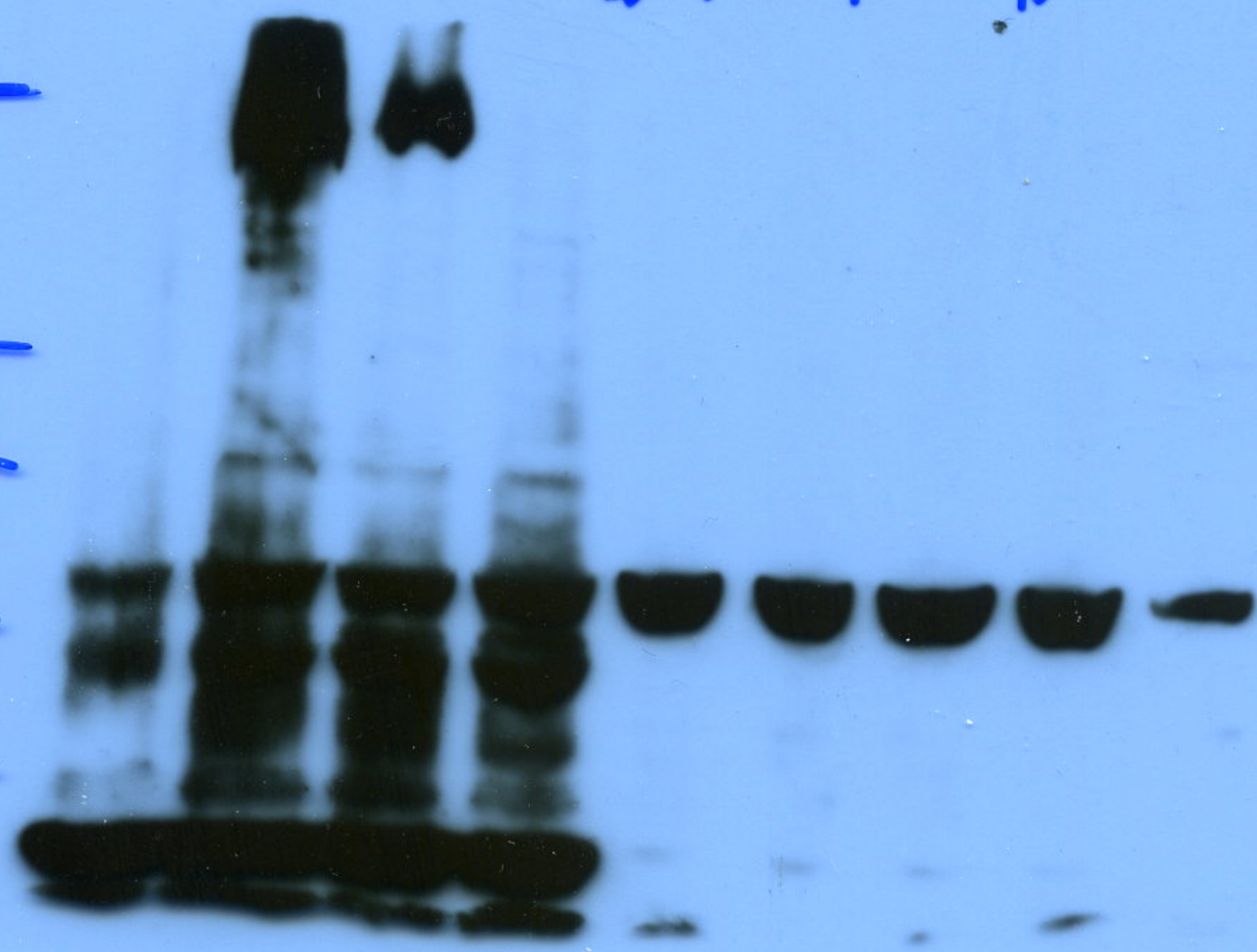

Actin  
(after GFP)

Supplement: Supplementary file 2 [file LSA-2020-00661_SdataFS2_2.pdf]

α Vav

120

95

78

liver

spleen

N

P

P

N

N

P

P

N

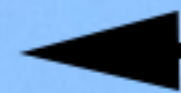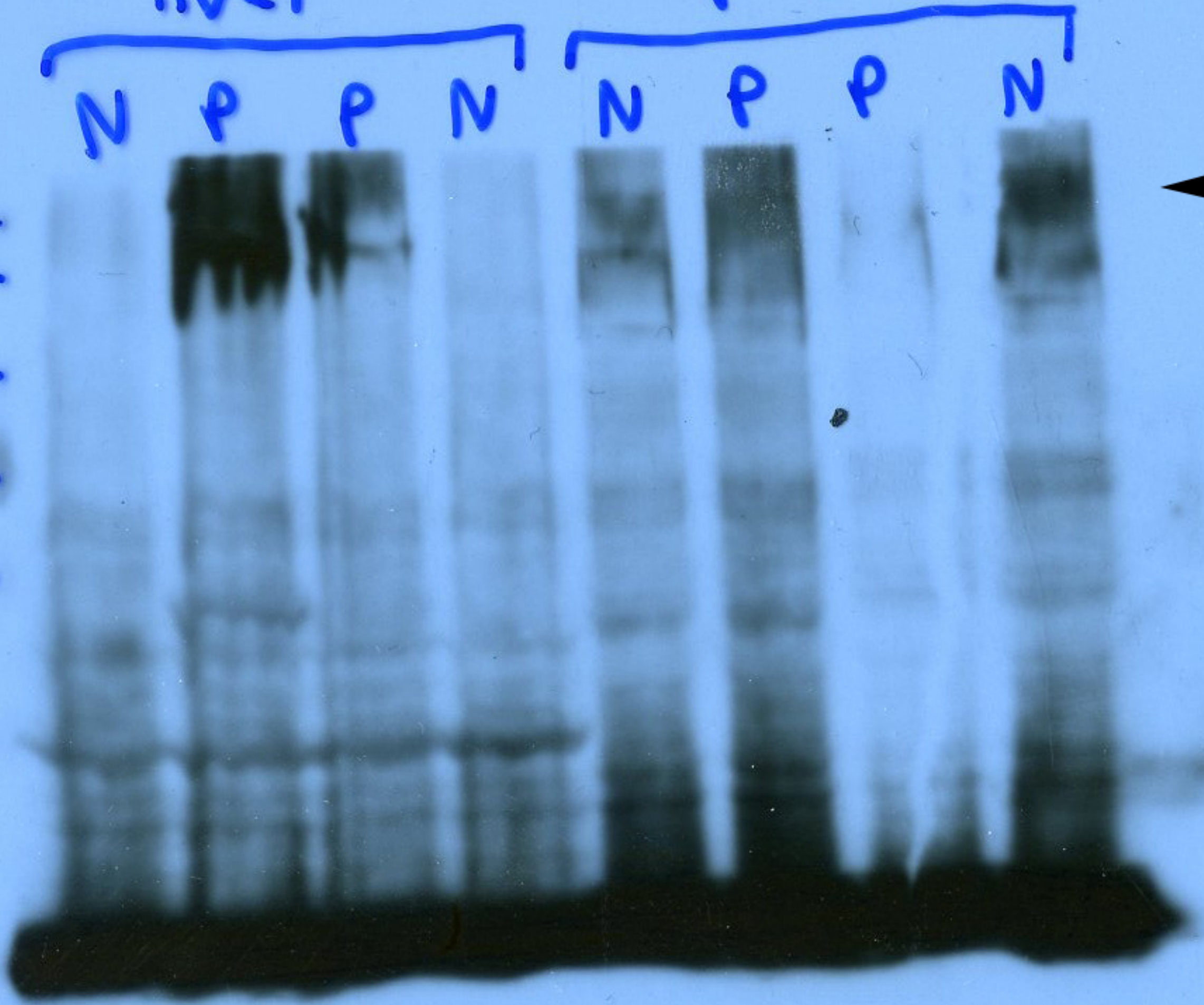

Supplement: Supplementary file 3 [file LSA-2020-00661_SdataFS2_3.pdf]

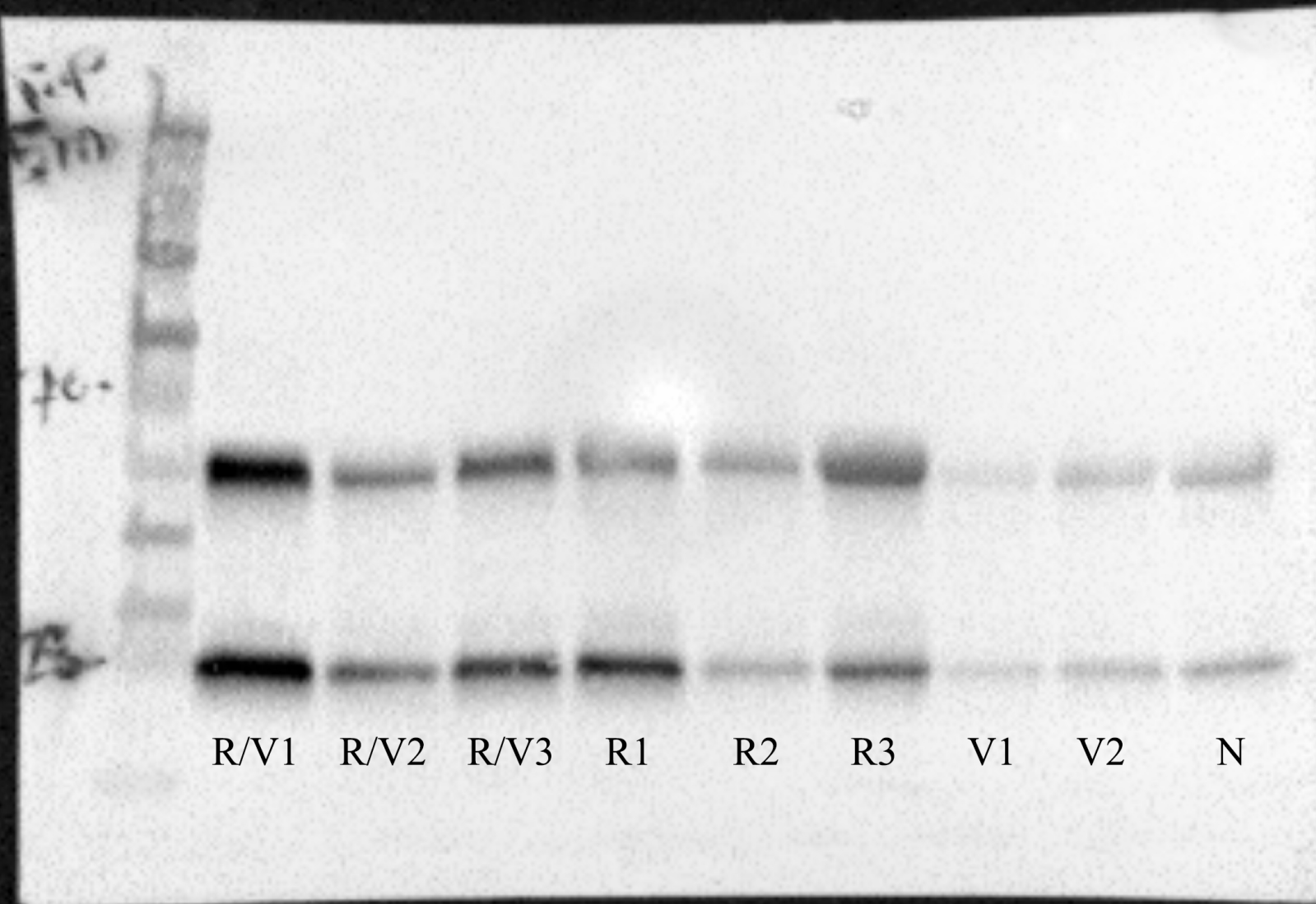

Supplement: Supplementary file 5 [file LSA-2020-00661_SdataF6A_1.pdf]

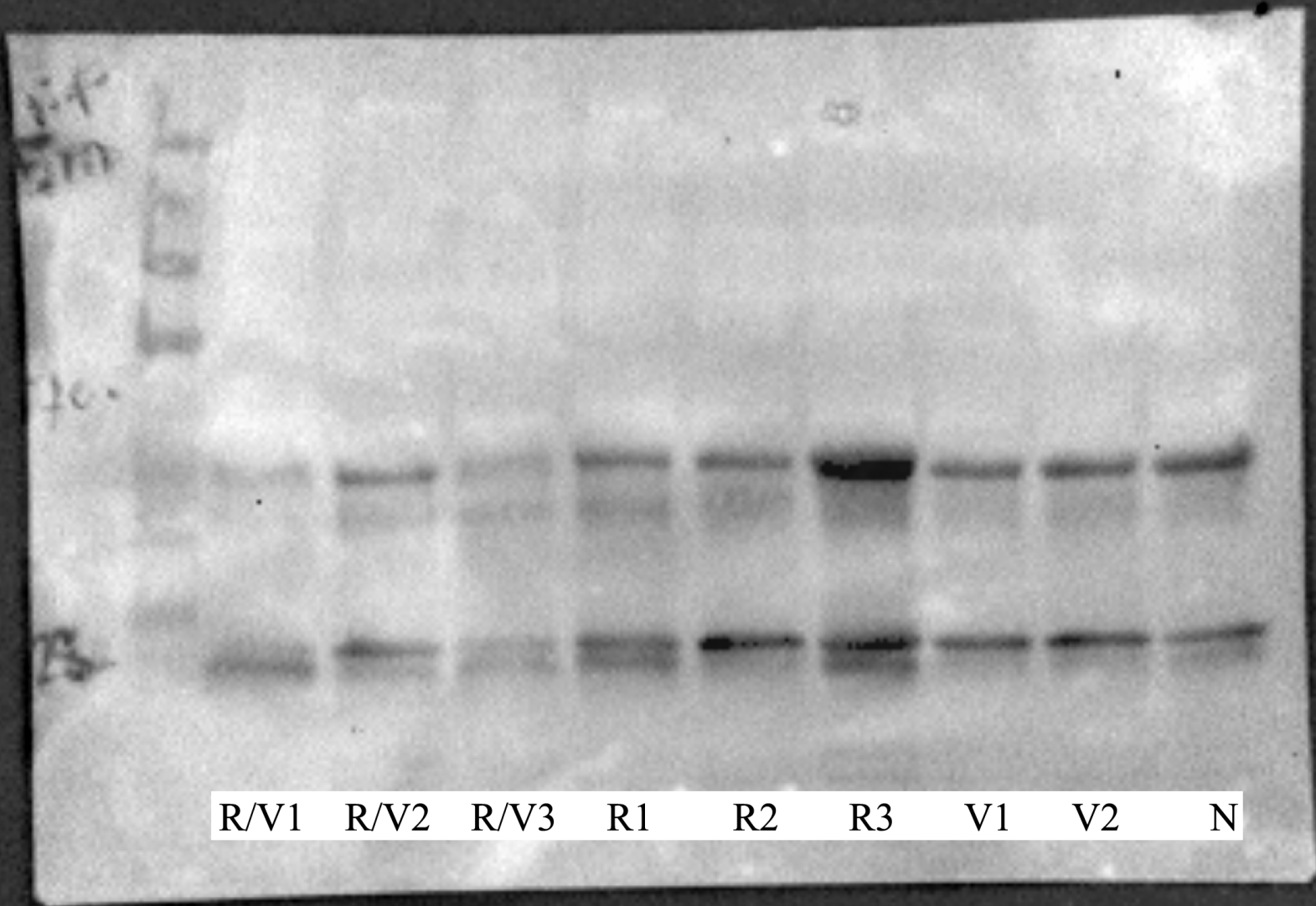

Supplement: Supplementary file 6 [file LSA-2020-00661_SdataF6A_2.pdf]

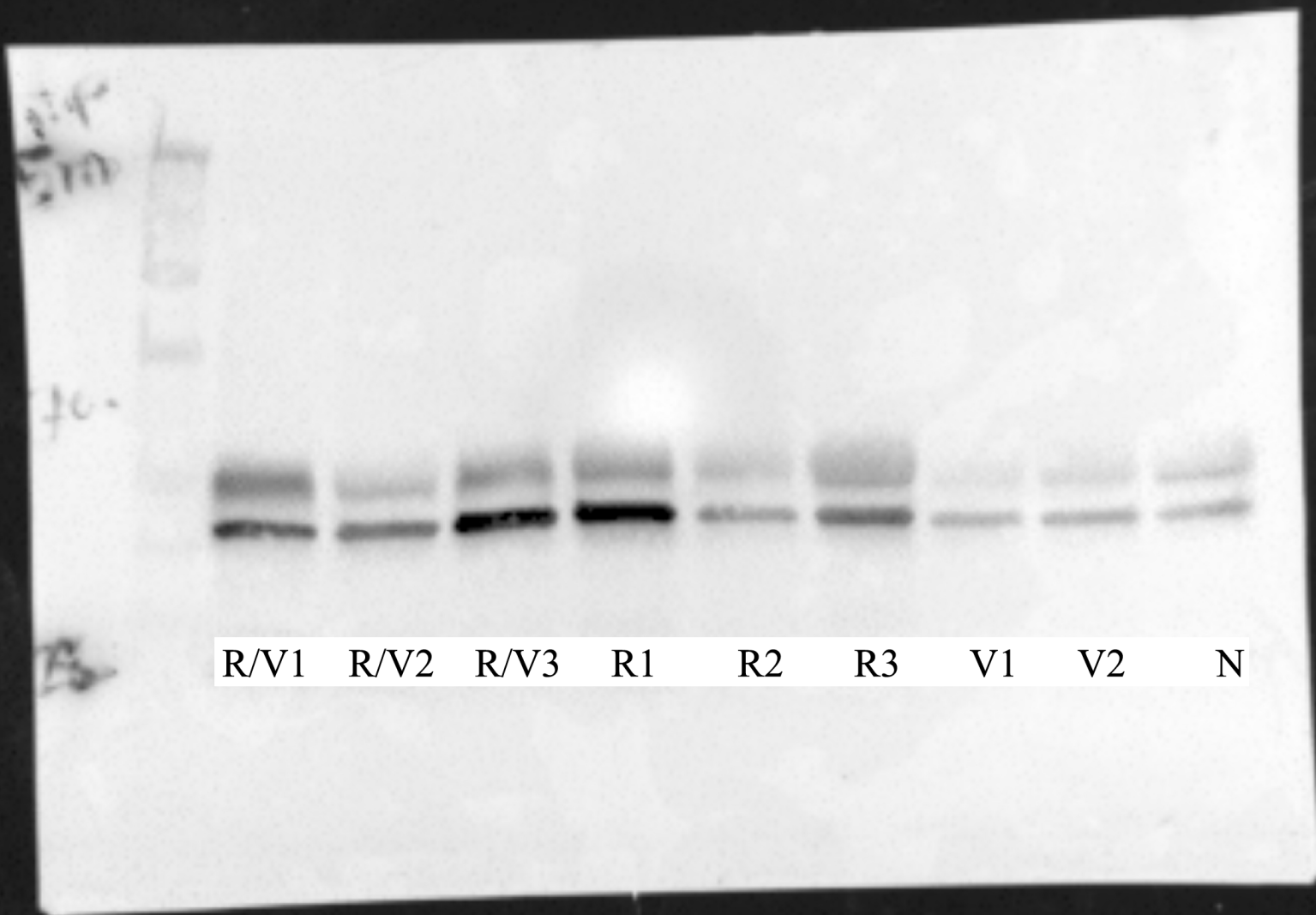

Supplement: Supplementary file 7 [file LSA-2020-00661_SdataF6A_3.pdf]
