# Supplementary material for: Vav1 and mutant K-Ras synergize in the early development of pancreatic ductal adenocarcinoma in mice
Source: Life Sci Alliance. 2020 Apr 10;3(5):e202000661. doi: 10.26508/lsa.202000661 (PMC7156281; doi:10.26508/lsa.202000661)
Supplement: Supplementary file 4 [file LSA-2020-00661_TableS1.docx]

**Supplementary Table 1.** *Number of mice used in various experiments.*

*Figure 1: Expression of GFP in pancreata from different mouse lines*

| Mice  Time points | Control | Vav1 | K-Ras^G12D^ | K-Ras^G12D^/Vav1 |
| --- | --- | --- | --- | --- |
| 1 month | 1 | 1 | 2 | 2 |
| 2 months | 1 | 1 | 1 | 1 |
| 3.5 months | 2 | 2 | 2 | 3 |
| 5 months | 2 | 3 | 1 | 3 |
| 12 months | 2 | 4 | 2 | 4 |

*Figure 4: Proliferation of pancreatic cells from control, Vav1, K-Ras^G12D^ and K-Ras^G12D^/Vav1 mice.*

| Mice  Time points | Control | Vav1 | K-Ras^G12D^ | K-Ras^G12D^/Vav1 |
| --- | --- | --- | --- | --- |
| 1 month | 4 | 4 | 4 | 7 |
| 2 months | 5 | 4 | 4 | 7 |
| 3.5 months | 5 | 6 | 7 | 11 |
| 5 months | 3 | 3 | 8 | 9 |
| 12 months | 4 | 5 | 5 | 7 |

*Figure 5: Relative Erk phosphorylation in malignant pancreatic lesions from different mice.*

| Mice  Time points | Control | Vav1 | K-Ras^G12D^ | K-Ras^G12D^/Vav1 | No. of experiments |
| --- | --- | --- | --- | --- | --- |
| 1 month | 2 | 4 | 3 | 4 | 3 |
| 2 months | 3 | 2 | 3 | 4 | 3 |
| 3.5 months | 3 | 2 | 3 | 3 | 4 |
| 5 months | 2 | 2 | 3 | 2 | 3 |

*Figure 6: Activation of Rac-GTP in malignant pancreatic lesions from K-Ras^G12^/Vav1 mice.*

| Mice  Time points | Control | Vav1 | K-Ras^G12D^ | K-Ras^G12D^/Vav1 | No. of experiments |
| --- | --- | --- | --- | --- | --- |
| 1 month | 2 | 4 | 3 | 4 | 3 |
| 2 months | 3 | 2 | 3 | 4 | 3 |
| 3.5 months | 3 | 2 | 3 | 3 | 4 |
| 5 months | 2 | 2 | 3 | 2 | 3 |
