# Supplementary material for: Vav1 and mutant K-Ras synergize in the early development of pancreatic ductal adenocarcinoma in mice
Source: Life Sci Alliance. 2020 Apr 10;3(5):e202000661. doi: 10.26508/lsa.202000661 (PMC7156281; doi:10.26508/lsa.202000661)
Supplement: Supplementary file 8 [file LSA-2020-00661_TableS2.docx]

**Supplementary Table 2: Primers used for transgenic mice genotyping.**

| Transgene | Primers |  |
| --- | --- | --- |
| *Ptf1aCreER* | Forward | 5' – TGCCACGACCAAGTGACAGC-3' |
|  | Reverse | 5'- CCAGGTTACGGATATAGTTCATG-3' |
| *LSLrtTA* | Rosa5: | ROSA5: 5'- GAGTTCTCTGCTGCCTCCTG-3' |
|  | RTTA3: | RTTA3: 5'- AAGACCGCGAAGAGTTTGTC-3' |
|  | ROSA3: | ROSA3: 5'-CGAGGCGGATACAAGCAATA-3' |
| *LSL-KRas^G12D^* | K-RAS1 | 5'-CTAGCCACCATGGCTTGAGT-3' |
|  | K-RAS2 | 5'-ATGTCTTTCCCCAGCACAGT-3' |
|  | K-RAS3 | 5'-TCCGAATTCAGTGACTACAGATG-3' |
| *TetO-Vav1* | Forward | Vav1 F: 5'-AGGGTGACATCATCAAGATCCTTAACAA-3' |
|  | Reverse | GFP-R: 5'-TGCAGATGAACTTCAGGGTCAGCTT-3' |
