# Supplementary material for: Vav1 and mutant K-Ras synergize in the early development of pancreatic ductal adenocarcinoma in mice
Source: Life Sci Alliance. 2020 Apr 10;3(5):e202000661. doi: 10.26508/lsa.202000661 (PMC7156281; doi:10.26508/lsa.202000661)
Supplement: Supplementary file 9 [file LSA-2020-00661_TableS3.docx]

**Supplementary Table 3.** *List of antibodies used in this study.*

| **Antibody** | **Application** | **Company** |
| --- | --- | --- |
| Anti Ki67 | IHC | ThermoScientific (SP6) |
| Anti Vav1 | IHC | Cell signaling (#2505) |
| Anti Vav1 | IF, WB | Millipore (#05-219) |
| Anti ERK | WB | Abcam (#Ab17942) |
| Anti pERK | IHC | Cell Signaling (#4370) |
| Anti pERK | WB | Millipore (#AW39R) |
| Anti-Pan-Cytokeratin | IHC | Dako (#Z0622) |
| Anti-GFP | IHC, IF, WB | Abcam (#Ab6673) |
| Anti-EGFR | IHC | Abcam (#Ab52894) |
| Anti-pEGFR | IHC | Cell Signaling 3777 |
| Anti Rac1-GTP | IF, WB | New East Biosciences (#26903) |
| Goat Anti-Mouse IgG (Alexa Fluor® 594) | IF | Invitrogen (Cat # A-21125) |
